# Hemidystrophic Thorax Mimicking Scoliosis

**DOI:** 10.2174/1874325001812010252

**Published:** 2018-07-19

**Authors:** Hans-Rudolf Weiss, Sarah Seibel

**Affiliations:** Physical Medicine and Rehabilitation, Chiropractor, Gesundheitsforum Nahetal, Alzeyer Str. 23, D-55457, Gensingen, Germany

**Keywords:** Hemidystrophic thorax, Thoracic deformities, Scoliosis, Scoliotic deformity, ATR, Cobb angle

## Abstract

**Background:**

We regularly use Angle of Trunk Rotation (ATR) measurements for scoliosis screening and also for clinical follow-up of our scoliosis patients under treatment. In some patients, when ATR measurements exceed the screening threshold but without a significant degree of curvature on the X-ray (Cobb angle), a Hemidystrophic Thorax (HDT) is diagnosed. The purpose of this paper was to present a case series of patients with this kind of thoracic deformity because this may be mimicking scoliosis to a significant degree.

**Materials and Methods:**

This case series is a consecutive series of patients where the first author detected a hemidystrophic thorax instead of or in combination with scoliosis. A 3D scan of the trunk was made and adjusted to the coordinates in order to achieve an upright orientation of the upper trunk. The scan was scaled in order to determine certain anatomic landmarks, as performed in preparation for bracing. The scan was cut horizontally at the xiphoid level and the plane at this level was analysed visually in order to detect deformations that were different to the typical scoliotic deformations in the horizontal plane.

**Results:**

Seven cases were analysed and described in more detail.

**Conclusion:**

The condition of HDT may lead to significant rib humps that mimic scoliosis. According to our case series, mild scoliosis can also be associated with HDT. HDT, according to the cases presented in this study, seems to be a relatively benign deformity. Long-term observations are necessary before a final conclusion can be drawn with respect to prognosis.

## 1 INTRODUCTION

Pectus Excavatum (PE), Pectus Carinatum (PC), Poland Syndrome (PS), Sunken Chest Deformity (SCD), Barrel Chest Deformity (BCD), Body Builder Deformity (BBD), and Long Upper Chest Wall (LCW) are Chest Wall Deformities (CWDs) that are documented in the literature [1].

PE is the most common congenital chest wall deformity, accounting for over 90% of all chest wall deformities [2]. PE, a deformity of the anterior chest wall, is characterized by a depression in the lower sternum and adjacent ribs. The deformity may be symmetrical or asymmetrical [3]. An infant may have the deformity at birth or develop it later during childhood [3].

The second most common deformity of the anterior chest wall is PC, also called pigeon chest. It is a diverse deformity and may be symmetric or asymmetric [4].

CWDs can be diagnosed using a 3D body scanner, CT scans or MRI [5, 6].

PS [7, 8] is a rare congenital anomaly described first in 1897 by Alfred Poland and is characterized by the partial or complete absence of the pectoralis major muscle, ipsilateral symbrachydactyly and is occasionally associated with other malformations of the anterior chest wall and breast.

Mild to moderate CWDs may be left without treatment or can be treated conservatively with different braces. The most important treatment indication is because CWD may affect the physical and psychological development of the patient [9].

Treatment of CWDs may be conservative [10, 11] or surgical [9]. Major adverse outcomes of chest wall surgery may be underreported [12].

Breast asymmetry is a common concern in young female patients with scoliosis. The majority of patients with significant scoliosis experience breast asymmetry of up to 21% in addition to their scoliosis-related CWD [13, 14].

**Fig. (1):**
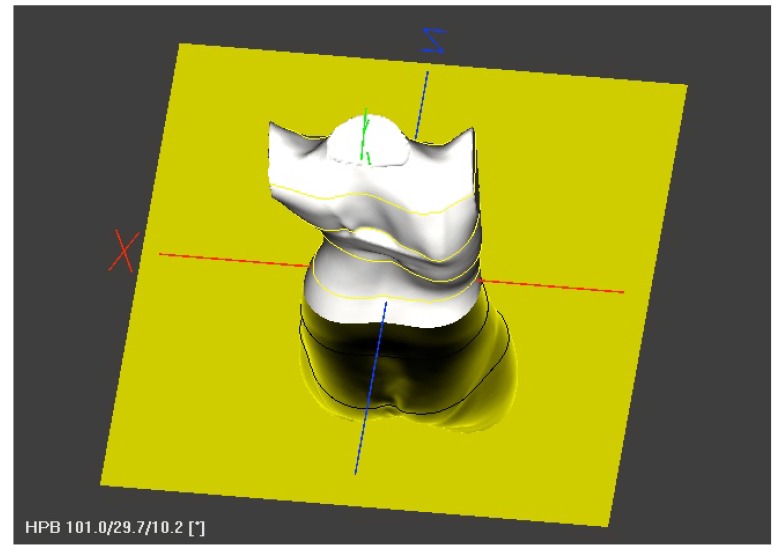
The scoliotic CWD is an expression of thoracic torsion due to scoliotic deformity of the spine and adjacent ribs. This is a case with severe thoracic scoliosis (65° Cobb) with a huge rib hump.

**Fig. (2):**
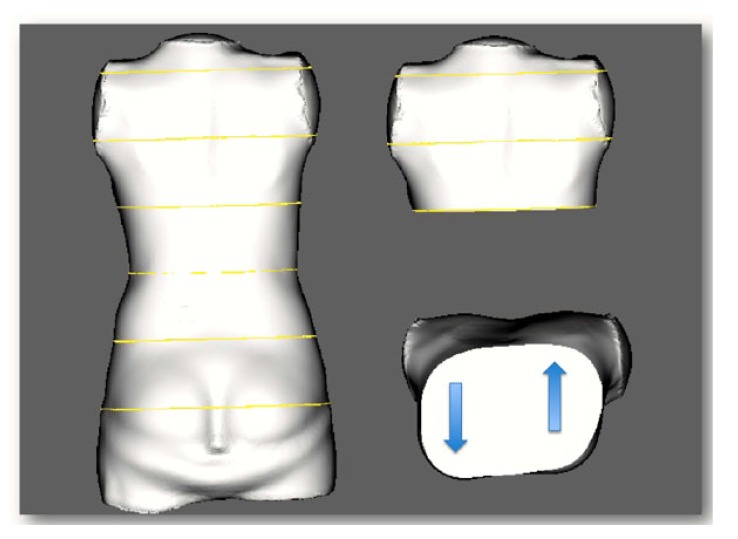
In thoracic scoliosis, we typically find a rotation of the trunk backwards on the rib hump side and a rotation forward on the thoracic concave side (see arrows). This is a case of thoracic scoliosis with a 40° Cobb.

A scoliotic CWD, unlike PE or PC, does not only concern the anterior chest wall but is an expression of thoracic torsion due to the scoliotic torsion of the spine and adjacent ribs (Fig. **1**). Typically, we find a rib hump dorsally on the convex side of the curvature when the thoracic spine is affected and a lumbar hump dorsally on the convex side of a lumbar curve when the lumbar spine is affected. When thoracic scoliosis is analysed more closely using a 3D scan of the trunk, we typically find a rotation of the trunk backward on the rib hump side and a rotation forward on the thoracic concave side (Fig. **2**).

**Fig. (3):**
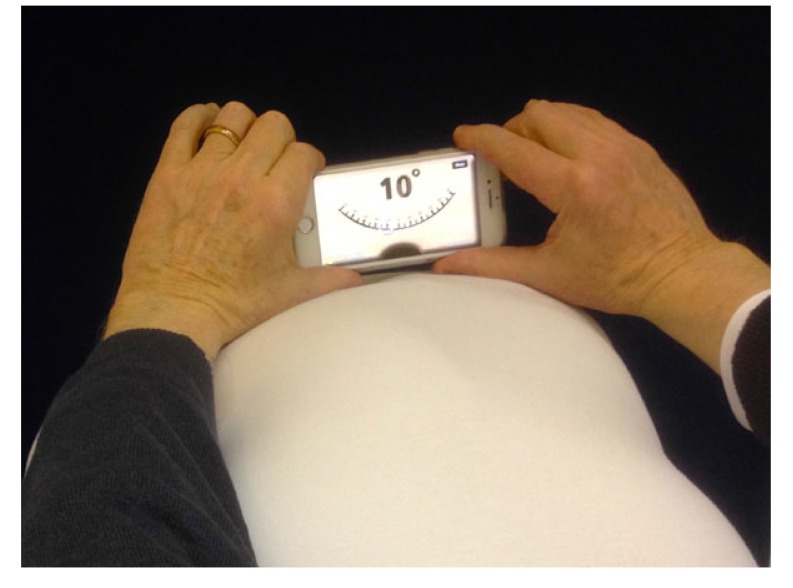
Some apps for mobile phones are available to measure the angle of trunk rotation (ATR)/angle of trunk inclination (ATI). These tools are also used in scoliosis screening.

The dorsal rib hump and the dorsal lumbar hump can be measured using a scoliometer (Orthopedic Systems Inc., Hayward, CA). Today, some apps for mobile phones are available to measure the angle of trunk rotation (ATR)/Angle of Trunk Inclination (ATI) (Fig. **3**) [15]. These tools are also used in scoliosis screening. The Scoliosis Research Society task force suggested that a measure between 5 and 7 degrees be used as the screening threshold [15, 16]. Beyond this threshold, an X-ray of the spine is indicated [16].

We regularly use ATR measurements for scoliosis screening and also for clinical follow-up of our scoliosis patients under treatment [17]. We have found in some patients ATR measurements that exceed the screening threshold but without a significant degree of curvature on the X-ray (Cobb angle). Therefore, the first author re-examined these patients more closely with respect to the ventral side of the thorax. In all of these patients, by inspection, the clinical picture was different to the typical scoliotic deformity. Different anterior/posterior measurements were found when the two hemithoraces were compared with each other. The purpose of this paper was to present a case series of patients with this kind of thoracic deformity because this may mimic scoliosis to a significant degree.

## 2 MATERIALS AND METHODS

All of the cases described here had been referred to the first author for suspicion of scoliosis. Neither in the patient histories nor during clinical inspection there were any signs of other malformations or syndromes (*e.g*. PS, Klippel-Trenaunay-Weber syndrome) evident. The dorsal rib hump was the only visible clinical sign. All patients otherwise seemed healthy.

This case series was a consecutive series of patients where the first author detected a hemidystrophic thorax (HDT) instead or in combination with scoliosis.

A 3D scan of the trunk was made and adjusted to the coordinates in order to achieve an upright orientation of the upper trunk. The scan was scaled in order to determine certain anatomic landmarks, as performed in preparation for bracing. The scan was cut horizontally at the xiphoid level and the plane of this level was analysed visually in order to detect deformations that were different to the typical scoliotic deformations in the horizontal plane (Fig. **2**).

## 3 CASES

**Fig. (4):**
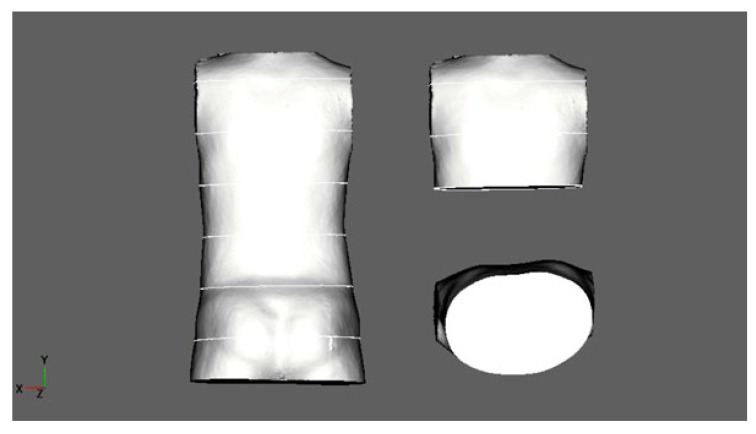
Scan of a 10-year-old boy with an ATR of 6–7 despite no significant lateral deviation clinically.

**Fig. (5):**
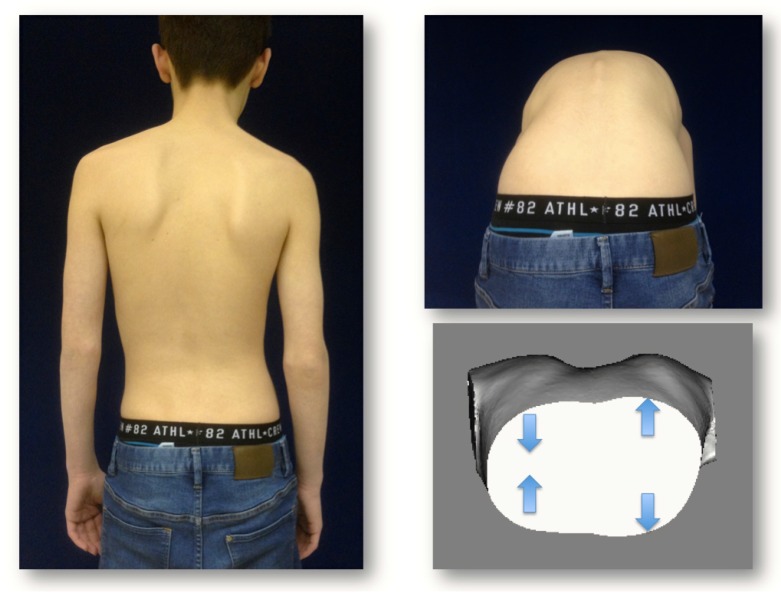
Scan and clinical picture including forward bending test of a 14-year-old boy with an ATR of 8° showing a typical hemidystrophic thorax.

**Fig. (6):**
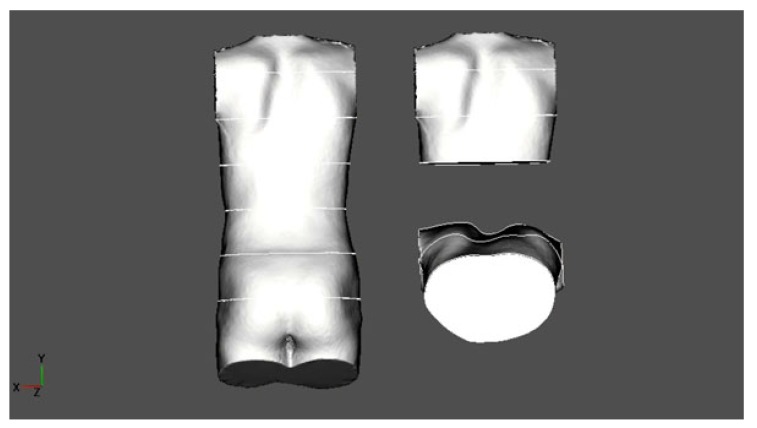
Scan of a 10-year-old girl with a thoracic ATR of 10° and a lumbar ATR of 2°.

**Fig. (7):**
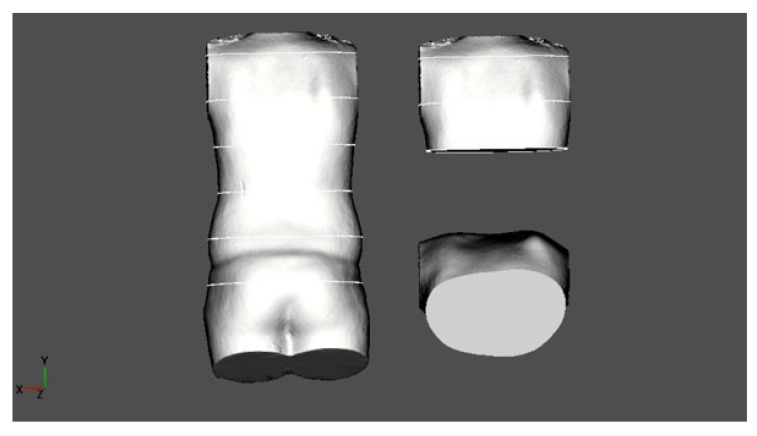
Scan of a 13-year-old boy with a thoracic ATR of 10° and a lumbar ATR of 0° and a hemidystrophic deformity of the thorax.

**Fig. (8):**
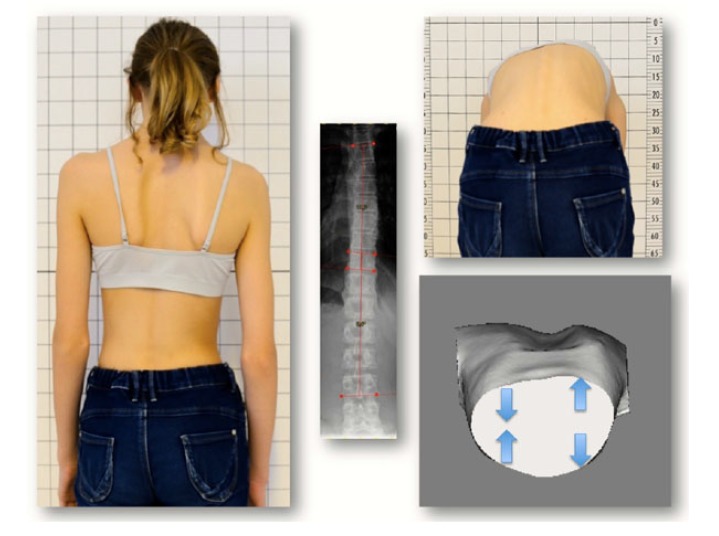
Scan and clinical picture including a forward-bending test of a 13-year-old girl with a thoracic ATR of 17° and 10° same side lumbar while the X-ray showed Cobb angles of only 11° for thoracic and 9° for lumbar.

(U.S.): A 10-year-old boy with an ATR of 6-7°; clinically, there was no significant lateral deviation. Diagnosis: Scoliotic posture, thoracic deformity diagnosed clinically. However, on the scan, no real HDT was diagnosed. Therefore, the final diagnosis was just a scoliotic posture. No X-ray was made (Fig. **4**).
(B.N.): A 14-year-old boy with an ATR of 5° at the first presentation (5/17). Clinically, there was a small lateral deviation with decompensation to the left but a rib hump on the right. Diagnosis: Scoliotic posture, HDT. No X-ray was made. There was an increase in ATR to 8° at the second presentation (9/17) but stable at the third presentation (1/18) when the pictures were made (Fig. **5**).
(J.J.): An 8-year-old girl presenting with an early onset scoliosis of 26° thoracic and 22° lumbar (9/16), a thoracic ATR of 10° and a lumbar of 5°. Brace treatment was initiated. The brace was outgrown quickly so a new night-time brace was prescribed on 1/17 with a Cobb angle of 18° thoracic and 11° lumbar. At the presentation on 8/17, there was no significant lateral deviation visible clinically nor in the Formetric surface topography scan. Therefore, a closer investigation of the thorax was performed clinically and an HDT was diagnosed. The thoracic ATR at that time was 11° and lumbar ATR was 3°. The brace treatment was commenced at this time. At the last presentation (12/17), the thoracic ATR was 10° and the lumbar was 2°. The 3D scan on the picture was made at this time (Fig. **6**).
(L.J.): An 11-year-old boy presenting with an early onset scoliosis of 20° thoracic curve and 10° lumbar curve (8/16). Thoracic and lumbar ATR were 10° and 2°, respectively. Physiotherapy was initiated as there was no sign of maturation yet (Tanner 1). After 13 months (9/17), thoracic and lumbar ATR had progressed to 13° and 3°, respectively. A new X-ray showed no progression of the curve with a Cobb angle of 17° for the thoracic curve and 9° for the lumbar curve. A night-time brace was initiated at that time because of an increasing rib hump. At the last follow-up (12/17), the thoracic ATR had reduced to 10° and the lumbar ATR to 0° and an HDT was diagnosed at that time (Fig. **7**).
(H.L.): An 11-year-old girl presenting with a scoliotic posture with a Cobb angle on the X-ray of 10° for the thoracic area and 10° for the lumbar area (4/16) and an HDT was diagnosed. Thoracic ATR was 10° initially. The condition was stable on 8/16. At the next presentation (2/17), the thoracic ATR had increased to 14° and the lumbar ATR to 5° on the same side as the thoracic. An X-ray was made showing a Cobb angle of 13° for the thoracic area and 18° for the lumbar area and Risser type 0. A night-time Chêneau-style CAD brace was prescribed. At the next follow-up (5/17), the thoracic ATR was significantly reduced to 8° and the lumbar ATR to 4°. At the follow-up on 9/17, ATR had increased again, despite brace wearing, to 10° and 7°. A short-term follow-up on 10/17 showed a stable condition. Finally on 1/18, the thoracic ATR had increased drastically to 17° and the same side lumbar ATR to 10° while the X-ray showed a thoracic Cobb angle of 11° and a lumber Cobb angle of 9° only (Fig. **8**).
(M.E.): The first presentation on 9/13 was described with a thoracic Cobb angle of 18° and a lumbar Cobb angle of 13° and a thoracic ATR of 9° and a lumbar ATR of 0°. Diagnosis: Early onset scoliosis starting at 7 years of age. A night-time brace was prescribed. There was a constant reduction over the next two years with an increase back to 8° thoracic at the age of 9 (8/15). A new night-time brace was prescribed and ATR reduced to 4° again. ATR increased to 8° by 3/17 with a new X-ray showing a Cobb angle of only 13°. HDT was detected on 6/17.

A follow-up was made over one year without bracing and ATRs were between 6 and 7°.

**Fig. (9):**
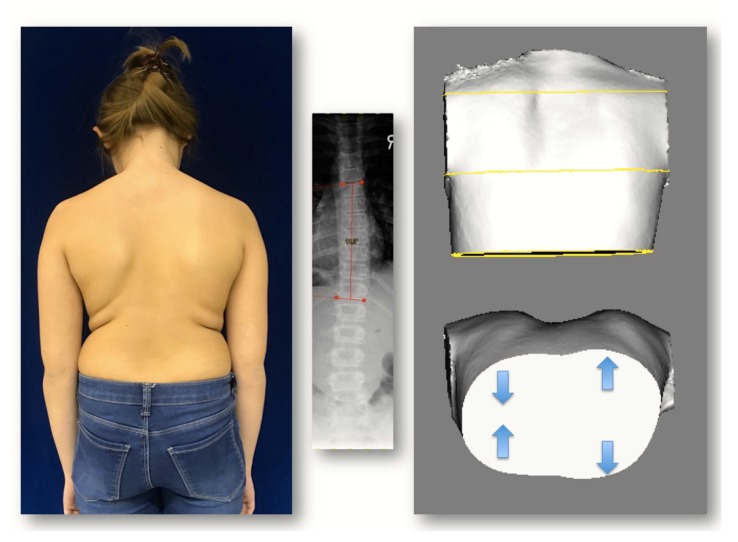
Scan and clinical picture of an adolescent girl with an ATR of 10° and a Cobb angle of 11° only. Therefore, no correlation was found between the development of the Cobb angle and of ATR.

On 2/18, an increase in ATR to 10° with a new X-ray showed a Cobb angle of only 11°. Therefore, no correlation was found between the development of the Cobb angle and of ATR (Fig. **9**).

**Fig. (10):**
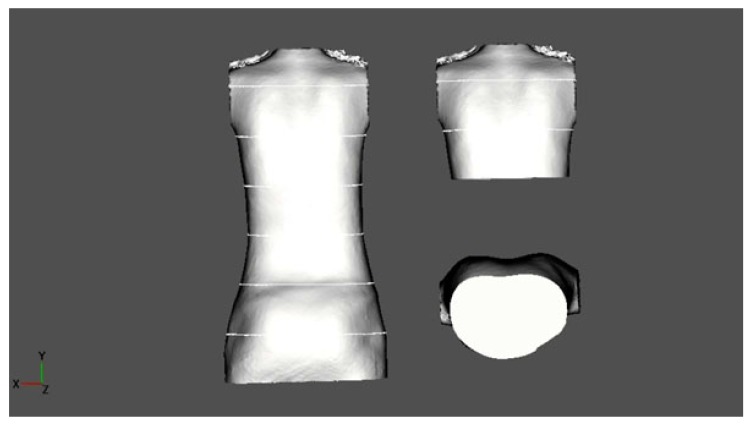
A nearly 13-year-old girl presented (2/18) with a thoracic ATR of 8° and a Cobb angle of 21° for the thoracic area and 17° for the lumbar area and Risser type 0.

Case 7 (S.R.): An almost 13-year-old girl presented (2/18) with a thoracic ATR of 8° and a thoracic Cobb angle of 21° and lumbar Cobb angle of 17°, Risser 0. Diagnosis: Scoliosis, thoracic deformity. Brace treatment was initiated. An HDT was diagnosed (Fig. **10**).

## 4 DISCUSSION

As has been shown in this case series, an HDT was found in patients with slight scoliosis and in cases without scoliosis (Cobb angle of 10° or less). The threshold of an ATR of 5-7° usually corresponds to a Cobb angle above 10° in the X-ray. In the experience of the first author, an ATR of 7° may correspond to a Cobb angle exceeding 20°, which in the immature patient, may be a bracing indication.

The curves of the HDT patients, however, have been shown to be relatively small. Only two patients from our series had a curve exceeding 20°. This fact makes it reasonable to assume that an HDT may lead to mild lateral deformities of the spine.

As HDT has not been found in major curves (> 30°) under brace treatment, we would not assume that the condition leads to severely progressive curves.

In one of our cases (Case 5), HDT led to a significant thoracic deformity with an ATR of 17°, which usually corresponds to a Cobb angle on the X-ray exceeding 45°. In this case, the first author is currently discussing with the patient and her mother about a special brace to address this deformity. In the mid-term, the other cases seem relatively benign.

While Ho *et al*. describe thickness variations between the left and right sides of the trunk at different regions including the thorax [18] a systematic description of HDT as to our knowledge has not been found as of yet. HDT seems to be a rare condition, which is not yet extensively described in the literature. Therefore, it seems important, especially for the scoliosis specialist, to know this condition in order to allow safe guidance of patients with this condition and avoid discussions about scoliosis surgery.

Within this small sample of patients with HDT, a vast progression of the thoracic deformity in combination with a simultaneous reduction in the spinal deformity was found in one case. More information and longer follow-up periods are needed. Only with the end growth results can final conclusions be drawn.

## CONCLUSION

The condition of HDT may lead to significant rib humps, which mimic scoliosis. According to our case series, mild scoliosis can also be associated with HDT. HDT, according to the cases presented in this study, seems to be a relatively benign deformity. Long-term observations are necessary before a final conclusion can be drawn with respect to prognosis.
